# The White Plane in Esophageal Surgery: A Novel Anatomical Landmark with Prognostic Significance

**DOI:** 10.3390/cancers17244005

**Published:** 2025-12-16

**Authors:** Vladimir J. Lozanovski, Timor Roia, Edin Hadzijusufovic, Yulia Brecht, Franziska Renger, Hauke Lang, Peter P. Grimminger

**Affiliations:** Department of General, Visceral and Transplantation Surgery, University Medical Center of the Johannes Gutenberg University Mainz, Langenbeck Str. 1, D-55131 Mainz, Germany; vladimir.lozanovski@unimedizin-mainz.de (V.J.L.);

**Keywords:** esophageal surgery, esophageal anatomy, thoracic duct, intraoperative landmark, surgical anatomy, white plane, Morosow’s ligament, chylothorax, esophageal cancer, robot-assisted surgery, minimally invasive surgery, da Vinci esophagectomy

## Abstract

**Introduction:** Identification of the thoracic duct (TD) is essential during esophageal surgery to reduce the risk of complications such as chylothorax. The clinical significance of the white plane, or Morosow’s ligament—a consistent anatomical landmark along the esophagus—remains poorly defined. **Methods:** A total of 166 patients undergoing robot-assisted minimally invasive esophagectomy (RAMIE) were analyzed. Intraoperative visualization of the white plane was documented. Patient demographics, tumor characteristics, postoperative complications, management strategies, hospital length of stay, and overall survival were assessed. Complication severity was graded using the Clavien–Dindo classification. The Kaplan–Meier and multivariable Cox regression analyses were used to evaluate prognostic factors, including BMI, ASA score, pneumonia, pT status, pN status, neoadjuvant and adjuvant therapy, and white plane visualization. **Results:** The white plane was visualized in 154 patients (92.8%). Postoperative complications, management strategies, hospital length of stay, and 30-/90-day in-hospital mortality did not differ between groups with visualized and not visualized white planes. Median overall survival was significantly longer in patients with a visible white plane (43.1 vs. 13.1 months; *p* = 0.0079). The multivariable analysis identified ASA classification, pT stage, pN stage, and adjuvant therapy as independent predictors of overall survival, whereas lymph node stage and adjuvant therapy were independent predictors of recurrence-free survival. **Conclusions:** The white plane is a distinct intraoperative anatomical structure that can be visualized in most RAMIE procedures. Its identification may assist in TD recognition and provides a framework for describing mediastinal anatomy, but further studies are needed to determine its impact on surgical standardization and patient outcomes.

## Simple Summary

Surgery for esophageal cancer is complex, and working in the correct anatomical layer is essential to avoid complications and improve outcomes. This study investigated a natural tissue layer—the “white plane”—as an anatomical landmark during minimally invasive and robot-assisted esophageal surgery. The study assessed how often this structure can be visualized and whether its visibility influences patient outcomes. The white plane was visible in more than 90% of patients and facilitated reliable identification of the thoracic duct, which can cause serious complications if injured or inadequately managed during resection. Patients in whom the white plane was clearly visible showed better long-term survival. These findings suggest that this anatomical landmark may enhance the safety, precision, and standardization of esophageal cancer surgery and provide valuable guidance for future surgical research.

## 1. Introduction

Esophageal carcinoma is a clinically relevant pathology [1,2,3]. Histopathologically, adenocarcinoma and squamous cell carcinoma are the predominant types, with a notable increase in adenocarcinoma incidence over recent years [3,4]. Therapeutic management is generally multimodal and depends on the patient’s general condition, tumor stage, and individual preference [3,4]. The standard approach for locally advanced disease is multimodal; however, radical esophagectomy is the cornerstone of treatment with 5-year survival rates up to 50% [5]. Regardless of the chosen approach (open, laparoscopic, or robot-assisted), the surgical goal is a radical resection and reconstruction of gastrointestinal continuity, and precise dissection along the correct anatomical plane is essential to minimize trauma and achieve adequate lymph node clearance. Minimally invasive techniques aim to reduce morbidity and promote faster recovery, particularly by lowering the risk of recurrent laryngeal nerve injury. Thus, a clear understanding of the mediastinal anatomy is crucial for balancing curability and safety, yet a standardized anatomical concept for this region is lacking.

The thoracic duct (TD) originates from the cisterna chyli and runs along the thoracic aorta as a main lymphatic pathway. Previous studies have shown that thoracic duct lymph nodes metastases occurred in approximately 7% of patients undergoing transthoracic esophagectomy with TD resection for esophageal squamous cell carcinoma [6,7]. When comparing TD-preserved and TD-resected groups, TD resection with extensive lymphadenectomy was associated with improved prognosis in cT1N0 squamous cell carcinoma. However, the independent survival effect of TD resection was not assessed in those studies. A recent study investigating TD resection in patients with locally advanced esophageal squamous cell carcinoma demonstrated that TD resection confers a survival benefit only in selected cases, showing a pathological response after neoadjuvant therapy [8]. Despite multiple studies examining its prognostic relevance, the benefit of TD resection remains controversial [8,9,10,11].

Beyond oncologic outcomes, the surgical description and management of the TD remain insufficiently explored. Identification of the TD is essential during esophagectomy, as unrecognized injury or inadequate clipping can result in chylothorax, infection, or the need for radiological or surgical intervention. Advances in laparoscopic and robotic systems now allow highly precise dissection and improved visualization of delicate anatomical structures that might otherwise remain undetected [12]. Interestingly, a membrane located ventral to the TD may serve as an important anatomical landmark for its localization. This anatomic landmark, known as the “white plane” or “Morosow’s ligament,” was first described macroscopically in 1961 [13]. In 2021, a visceral sheath was identified dorsal to the esophagus and ventral to the TD, corresponding to Morosow’s earlier observations [14,15]. However, the potential surgical implications of this membrane have not yet been investigated. Based on embryological knowledge, a hypothetical model of the surgical anatomy of the mediastinum was developed, characterized by three features: a concentric and symmetrical three-layer structure, bilateral vascular distribution, and an interlayer potential space composed of loose connective tissue or “fascia”—a generic term for any fibrous connective tissue that covers or separates soft tissues and organs [16]. According to this model, the visceral layer, or the central core of the concentric structure contains the esophagus, trachea, thyroid gland, and recurrent laryngeal nerves. In this model, the visceral layer forms the central core containing the esophagus, trachea, thyroid gland, and recurrent laryngeal nerves; the vascular layer includes the aorta, vagal and phrenic nerves, and the TD; and the parietal layer comprises the vertebrae, sternum, ribs, skeletal muscles, and sympathetic nerve trunk [16]. The loose connective tissue forming the interlayer potential space is neutral to the three layers, as its attachment depends on the small gap created during surgical dissection. This space is not necessarily avascular, since peripheral vessels, nerves, and lymphatics traverse it between layers [16,17,18]. Consequently, this interlayer potential space can be difficult to delineate and dissect, particularly in bilateral regions where nerves and vessels transition between layers, whereas recognizing these layers may facilitate effective lymph node dissection around the recurrent laryngeal nerves and improve visualization of the TD [16].

This study aimed to evaluate the visibility and clinical relevance of the white plane as natural anatomical landmark and a potential dissection plane during robot-assisted minimally invasive esophagectomy (RAMIE), providing grounds for improved intraoperative identification of the TD during minimally invasive esophagectomies.

## 2. Patients, Materials, and Methods

This retrospective observational study included all complete intraoperative video records of patients who underwent RAMIE for confirmed esophageal carcinoma using the Da Vinci robotic system (Intuitive Surgical Inc., Sunnyvale, CA, USA) at the University Medical Center Mainz between May 2017 and April 2024. All RAMIE procedures were performed by a consistent surgical team within a single institution, ensuring the standardization of the procedure. Data were prospectively collected and systematically documented in an institutional database. Comprehensive follow-up was performed for all patients, ensuring complete outcome assessment. All patients provided informed consent permitting anonymous data and follow-up collection, with potential use for scientific analysis. Cases with missing or incomplete intraoperative video documentation were excluded.

### 2.1. Clinical, Pathological, and Survival Outcomes

The primary endpoint was the intraoperative identification of the white plane during RAMIE and its association with clinical, pathological, and survival outcomes.

Demographic and perioperative characteristics (age, sex, American Society of Anesthesiologists Physical Status Classification System (ASA), and body mass index (BMI), including BMI categories), pathological findings (tumor stage [pT], lymph node stage [pN], distant metastases [pM], histological subtype, Union for International Cancer Control (UICC) staging classification, and R classification), and the localization of R1 margins were also analyzed. BMI categorization was based on WHO thresholds, which correspond to the German national guideline values. Postoperative morbidity was evaluated for complications such as chylothorax, pneumonia, anastomotic leakage, and acute respiratory distress syndrome (ARDS), with severity graded according to the Clavien–Dindo classification. Management strategies for chylothorax were also analyzed, including thoracic drainage and fat-restricted diets, along with interventional approaches (pleurolysis, TD clipping, or lymphangiographic embolization).

In-hospital outcomes comprised general ward and intensive care unit (ICU) length of stay, as well as 30- and 90-day in-hospital mortality and recurrence. The outcome analysis included overall survival, evaluated using Kaplan–Meier curves and multivariable Cox regression. Covariates in the survival model were BMI, ASA score, pneumonia, pT, pN status, and neoadjuvant and adjuvant therapy to assess the independent prognostic value of white plane visualization.

#### 2.1.1. RAMIE: Thoracic Phase in Left Lateral Decubitus Position

The thoracic phase of the subtotal esophagectomy was performed in a left lateral decubitus position with radical mediastinal lymph node dissection for esophageal cancer [12]. The TD was either directly visualized or identified using indocyanine green (ICG) fluorescence guidance, which we routinely apply during this procedure. Following the introduction of the concentric-structured model by Fujiwara et al., emphasis was placed on understanding this layered anatomy to visualize the white plane and guide precise dissection of the TD, which allowed consistent identification of the duct within the vascular layer [16]. ICG was used to confirm TD visualization and to clarify cases in which the white plane was not identifiable.

Outcomes were compared between cases with and without a visible white plane, and the validity of the proposed concentric-structured model was assessed through an analysis of intraoperative images and video documentation.

#### 2.1.2. Video Material Evaluation

Postoperative video evaluation was performed by VJL, TR, and EH. Video evaluation was performed using the freeware “VLC media player version 3.0.21” software, followed by structured documentation in an institutional database. Visualization of the white plane, its clearest visualization captured on screenshot, the visualization and clipping of the TD before transection, and situs after duct transection were documented. Inter-rater reliability was evaluated for three observers who independently assessed the visibility of the white plane as a target structure using a binary scale. Pairwise agreement between raters was quantified using Cohen’s kappa (κ), and overall multi-rater agreement was assessed using Fleiss’ κ.

#### 2.1.3. Statistical Analysis

For categorical variables, absolute and relative frequencies were calculated. For metric variables, the mean, standard deviation, minimum, maximum, case numbers, and percentiles were determined. Statistical analyses were performed using R software, version 4 (R Development Core Team, 2018, https://www.r-project.org).

For comparisons between two groups with continuous variables, the Mann–Whitney U test was used. For comparisons involving more than two groups, the Kruskal–Wallis test was applied. For categorical data, the Chi-square test and, in the case of small sample sizes, Fisher’s exact test was performed. Overall survival (OS) was calculated using the Kaplan–Meier method. Survival curves were compared using the log-rank test, and multivariate survival analysis was conducted using the Cox proportional hazards model. A *p*-value < 0.05 was considered statistically significant.

## 3. Results

A total of 166 patients who underwent RAMIE were included in the analysis. The concentric layer structure was clearly demonstrated intraoperatively by dissecting from the dorsal side of the esophagus along the potential space between the visceral and vascular layers, containing loose connective tissue. This white plane was identified in 154 cases (93%), while it was not visible in 12 cases (7%). The white plane was identified as a distinct, avascular layer of loose connective tissue located just below and running parallel to the azygos vein. During video review, this anatomical layer was evaluated frame-by-frame based on its pale coloration, characteristic tissue texture, and consistent spatial relationship to surrounding structures. Agreement between raters was consistently high. Cohen’s κ indicated substantial agreement between R1 and R2 (κ = 0.74) and between R2 and R3 (κ = 0.74), while R1 and R3 showed perfect agreement (κ = 1.00). Fleiss’ κ for all three raters was 0.83, reflecting almost perfect overall reliability. Disagreements were mainly limited to cases in which R2 rated the structure as not visible while R1 and R3 rated it as visible; the remaining cases showed unanimous agreement.

**Figure 1:**
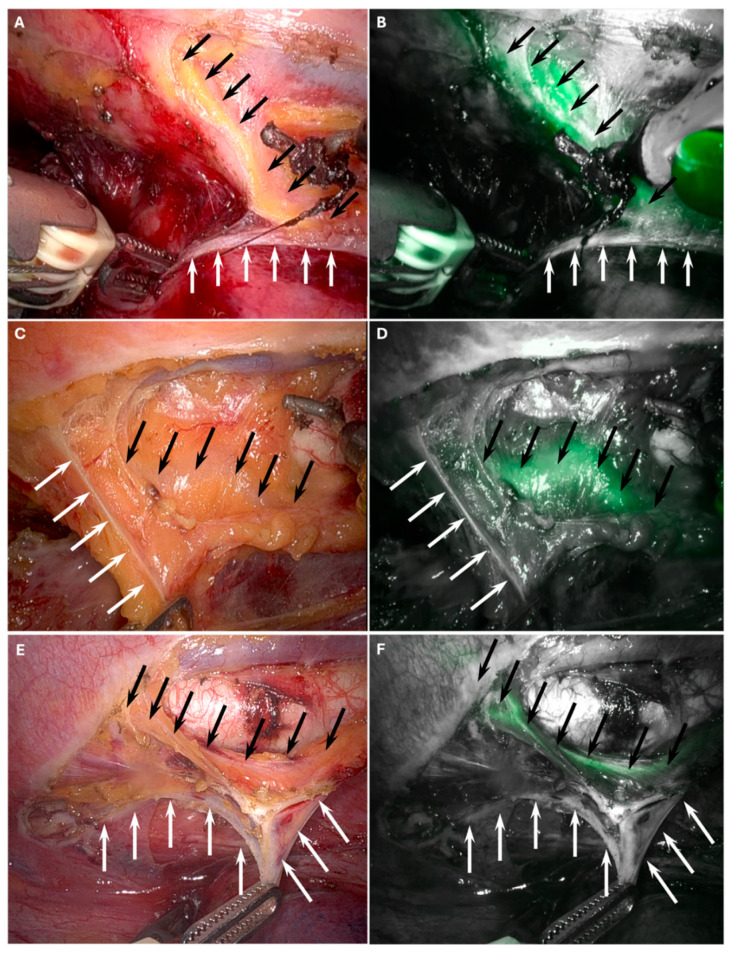
Intraoperative visualization of the white plane (white arrows) that delineates the viscero-vascular space, representing the appropriate surgical dissection plane and thoracic duct (black arrows). Panels (**A**,**C**,**E**) show the white plane and the thoracic duct during RAMIE. Panels (**B**,**D**,**F**) demonstrate the white plane and the thoracic duct with indocyanine green (ICG) fluorescence guidance.

Intraoperative views of the white plane and the TD, both without and with ICG fluorescence guidance, are presented in Figure 1.

The mean age of the cohort was 64 years, with no significant difference between the two groups (*p* = 0.924). Most patients were classified as ASA II or ASA III. However, no significant difference was observed regarding the ASA classification between the groups (*p* = 0.149). The mean BMI was 26.7 kg/m^2^, again without a significant intergroup difference (*p* = 0.816). BMI categorization showed a difference between the two groups: patients with a visible white plane more frequently exhibited normal weight status, while overweight patients with a BMI ≥ 25 predominated in the group without white plane visualization (*p* = 0.038) (Table 1).

No significant difference was found between groups regarding pT stage (*p* = 0.380). In contrast, pN status differed significantly: patients with a visible white plane showed a lower frequency of lymph node-positive stages (pN0) (*p* = 0.040). No significant differences were observed for distant metastases (pM) (*p* = 1.000), number of positive lymph nodes (*p* = 0.962), or R classification (*p* = 1.000). The location of the R1 resection margin (if present) was predominantly circumferential in patients with a visualized white plane (eight cases), though this finding was not statistically significant (*p* = 1.000) (Table 1).

Likewise, no statistically significant difference was observed concerning the UICC staging classification (*p* = 0.324). The histological subtype distribution was nearly homogeneous between groups (*p* = 1.000), with adenocarcinoma being predominant (80.1%) in both groups of patients (Table 1).

### 3.1. Complications and Management

Most of the patients (53%) experienced no postoperative complications (Table 2).

Regarding postoperative complications, particularly chylothorax with an overall incidence of 6%, no significant association was found with the presence of the white plane (*p* = 1.000). The same applied to other relevant complications such as pneumonia (*p* = 0.233), anastomotic leakage (*p* = 1.000), and acute respiratory distress syndrome (ARDS) (*p* = 1.000) (Table 3).

Similarly, no significant differences were observed in the severity of complications according to the Clavien–Dindo classification (*p* = 0.753) (Table 2). Active management was necessary in ten cases (6.0%) of patients with chylothorax, most commonly consisting of a combination of thoracic drainage and a fat-restricted diet (five patients; 3.0%). More complex interventions, such as pleurolysis, clipping or suturing of the TD, or lymphangiography with embolization, were applied in the remaining five cases (Table 4).

### 3.2. Operative Duration and Hospital Length of Stay Analysis

**Figure 2:**
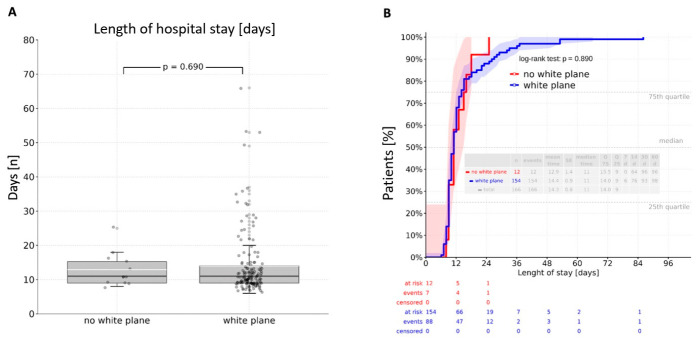
Length of hospital stay: (**A**)—direct comparison resulting in a mean value of 14,3 days in the normal ward without a significant difference between the groups (*p* = 0.690); (**B**)—log-rank analysis revealing an insignificant difference between the groups (*p* = 0.890).

**Figure 3:**
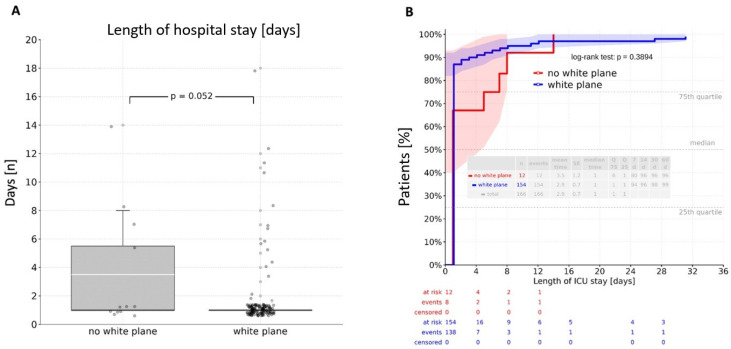
Length of stay at the intensive care unit: (**A**)—direct comparison resulting in a mean value of 2.9 days without a significant difference between the groups (*p* = 0.052); (**B**)—log-rank analysis revealing an insignificant difference between the groups (*p* = 0.389). ICU—intensive care unit.

Intraoperative visualization of the white plane did not prolong operative duration (*p* = 0.49). There was no significant difference between patients without white plane visualization and those with a visible white plane regarding both general ward and ICU stays. Direct group comparison revealed a mean stay of 14.3 days in the general ward and 2.9 days in the ICU, with no significant differences observed (*p* = 0.690; *p* = 0.052) (Figure 2A and Figure 3A). A cumulative analysis of general ward length of stay using the log-rank test yielded a *p*-value of 0.890 (Figure 2B), while the cumulative ICU stay comparison resulted in a *p*-value of 0.389 (Figure 3B).

### 3.3. Survival Analysis

**Figure 4:**
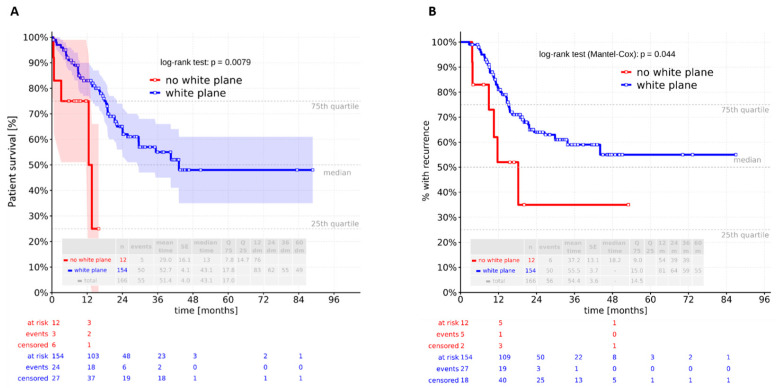
Kaplan–Meier analysis revealed a significant difference in (**A**) overall and (**B**) recurrence-free survival between the two groups (log-rank test: *p* = 0.0079 and 0.044).

No relevant differences in in-hospital mortality were observed between the groups. The 30-day in-hospital mortality rate was 0%, and the 90-day rate was 1.2% (*n* = 2), with both deaths occurring in the white plane group; however, this difference was not significant (*p* = 1.000). Similarly, overall mortality showed no intergroup difference (*p* = 0.535). In contrast, a Kaplan–Meier analysis revealed a significant difference in overall survival (log-rank test: *p* = 0.0079) (Figure 4A). Patients with a visible white plane had a median survival of 43.1 months, compared to 13.1 months in those without. The multivariate Cox analysis demonstrated that overall survival was independently influenced by ASA classification, pT stage, pN stage, and adjuvant therapy after adjustment for BMI (kg/m^2^), ASA score, pneumonia, pT status, pN status, and neoadjuvant and adjuvant therapy. The white plane was not confirmed to be an independent predictor of overall survival (*p* = 0.055) (Table 5).

Recurrence-free survival differed significantly between patients without a visible white plane and those with a visualized white plane (log-rank test: *p* = 0.044) (Figure 4B). In the recurrence-free survival analysis, only lymph node stage and adjuvant therapy remained independent predictors after adjustment for BMI, ASA score, pneumonia, pT status, pN status, and neoadjuvant and adjuvant therapy. The white plane was not an independent predictor in this model (Table 6).

## 4. Discussion

Modern laparoscopic and robotic systems further enable precise dissection and enhanced recognition of fine anatomical details that might otherwise remain undetected [12,19]. Still, the visualization of the TD is a frequently underrepresented aspect of esophageal surgery. The concentric-structured model proposed by Fujiwara et al. improved intraoperative awareness of the vascular and visceral layers and the structures within these layers [16]. These advances are clinically relevant, as minimally invasive esophagectomy aims to minimize surgical stress and morbidity while promoting faster recovery. The present study demonstrates that the Da Vinci robotic system allows for the visualization of the white plane that served as an anatomical guide to the TD. The results indicate that the intraoperative identification of the white plane during RAMIE is a reproducible feature that may be associated with improved oncologic outcomes without affecting perioperative morbidity or recovery.

The white plane was first described macroscopically in 1961 and later confirmed histologically as a visceral sheath located dorsal to the esophagus and ventral to the TD [13,14,15]. Meyer et al. described an interpleural ligament connecting the mediastinal pleura at the dorsal side of the esophagus [13]. Riddell et al. and Weijs et al. identified an aorto-esophageal ligament linking the esophagus and descending aorta on imaging and histology [20,21]. Tokairin et al. confirmed a thin membranous connective tissue between the esophagus, TD, azygos vein, and aorta, supporting Meyer’s macroscopic findings [14]. Fujiwara et al. developed a mediastinal model, defined by a concentric three-layer structure, bilateral vascular distribution, and an interlayer potential space composed of loose connective tissue or “fascia” [16]. The interlayer potential space, whose attachment varies with the dissection plane, is not entirely avascular, as peripheral vessels, nerves, and lymphatics traverse it. Consequently, this space can be difficult to delineate, particularly where nerves and vessels cross layers, while the recognition of these layers may facilitate lymph node dissection around the recurrent laryngeal nerves and improve TD visualization [16].

In the context of RAMIE or conventional minimally invasive esophagectomy (MIE), total mesoesophageal excision has been proposed to enhance local tumor control [22]. The mesoesophagus has been described in the middle and lower mediastinum, analogous to the dorsal mesentery of the abdominal digestive tract [23]. This structure, located below the tracheal bifurcation and sometimes termed the ‘aorto-esophageal ligament,’ corresponds to the dorsal blood supply from the descending aorta via the esophageal proper or left gastric artery [16].

En bloc tumor and lymph node resection is standard; however, routine TD resection is not recommended, and prophylactic ligation does not prevent postoperative chylothorax [8,9,10,11,22]. On the other hand, metastatic tumor cells can be present within the TD; therefore, some authors recommend its resection with adjacent tissue during total mesoesophageal excision [24,25,26,27]. Schurink et al. found TD lymph nodes in 86% of cadaveric specimens, suggesting potential lymphatic spread [26]. Some studies have linked TD resection to poorer outcomes and higher recurrence in esophageal squamous cell carcinoma [28,29]. However, TD resection may cause physiological disturbances, including chylothorax, altered immune and hemodynamic function, and impaired nutrient absorption [30]. Its role in patients with liver cirrhosis remains unclear, and some studies have linked TD resection to poorer outcomes and higher recurrence in esophageal squamous cell carcinoma. Therefore, routine TD resection remains controversial, and the recognition of natural landmarks and fascial planes is essential for safe radical dissection [24].

Technically, RAMIE allows optimal exposure and precision for selective TD dissection when indicated [30,31]. The advent of robotic surgery has refined mediastinal anatomy visualization, including periesophageal fasciae and vagal branches [21]. Although identification of the TD can be challenging after neoadjuvant therapy, ICG fluorescence and imaging enable real-time navigation and landmark visualization, facilitating total mesoesophageal excision. In contrast, such tools are not universally available in MIE, making recognition of the white plane under standard vision particularly valuable.

In this study, the white plane was identified in most RAMIE procedures, and its recognition is likely feasible during MIE as well. Its visualization was independent of age, ASA classification, or mean BMI but correlated with BMI category—being clearer in normal-weight patients and harder to distinguish in overweight individuals, likely due to excess periesophageal fat. However, this association should be interpreted cautiously as visualization was still achieved in many patients with BMI ≥ 30. BMI was evaluated both continuously and categorically: while mean BMI did not differ between groups, a higher proportion of overweight individuals was present in the non-visualized cohort. This likely reflects the small size of the non-visualized subgroup and suggests that categorical differences may arise from subgroup variability rather than a true effect on white plane detectability.

Postoperative outcomes were comparable regardless of white plane presence. Major complications (Clavien–Dindo ≥ 3b), including chylothorax, pneumonia, and anastomotic leakage, were rare and did not significantly differ between patients without white plane visualization and those with a visible white plane. Most management strategies were conservative, invasive interventions were infrequent, and hospital stays and mortality did not differ, indicating that white plane visualization does not affect perioperative recovery. Patients with a visible white plane demonstrated significantly longer overall and recurrence-free survivals. However, these associations did not persist after adjustment for BMI, ASA score, pneumonia, pT and pN stage, and treatment modalities. In the multivariate analysis of overall survival, ASA classification, pT stage, pN stage, and adjuvant therapy emerged as independent predictors, confirming that underlying tumor biology and patient-related factors remain the dominant determinants of survival. The white plane was not confirmed as an independent predictor; however, the *p*-value of 0.055 likely reflects the very small size of the non-visualized cohort rather than a true lack of effect and therefore warrants further evaluation. For recurrence-free survival, only lymph node status and adjuvant therapy remained independent predictors. The differences between the groups may reflect individual tumor biology and how patients respond to therapy. However, these findings should be interpreted with caution and regarded as exploratory, given the small number of patients without a visible white plane. Future studies are needed to better understand these effects and to determine whether the white plane has true prognostic value.

The limitations of this study include its retrospective, single-center design and the small number of patients without a visible white plane, which may restrict generalizability and reduce statistical power. The study was conducted in a high-volume robotic center, and reproducibility as well as white plane visibility may depend on surgical expertise, emphasizing the importance of understanding the concentric-structured layered anatomy to facilitate recognition and guide precise TD dissection. Our results demonstrate reproducible inter-rater reliability for the visibility assessment and provide confidence that the assessments reflect a stable and consistent evaluation process. Case observation and careful attention to this anatomy may help replicate these findings across different surgical platforms. Another limitation is the lack of analysis of the association between visceral and subcutaneous fat distribution and white plane visualization, which should be addressed in future studies. Furthermore, the white plane was identified based on surgical anatomy and visual characteristics; histopathologic validation was not performed, and potential inter-individual variability should be explored in future research. The study also did not analyze lymph node harvest or margin positivity rates stratified by white plane visibility—histological correlation should be assessed in subsequent studies. Improved overall and recurrence-free survivals in the white plane group could imply sampling variability or confounding by non-oncologic factors and should be interpreted with caution. Potential interactions between white plane presence, competing mortality risks (e.g., non-oncologic mortality), and TD management were not evaluated. The study primarily aimed to demonstrate that the white plane can be visualized in a larger patient cohort, and comparisons between RAMIE and MIE were beyond its scope. Finally, intraoperative identification was not independently validated, introducing the possibility of observer bias.

## 5. Conclusions

In conclusion, the white plane may represent an underappreciated anatomical landmark in MIE and RAMIE. Its identification likely reflects precise identification of the TD. Further prospective studies are warranted to clarify its clinical and prognostic relevance.

## Figures and Tables

**Table 1:** Patient collective.

		Total		WP Not Visualized		WP Visualized		*p*
		166		12 (7%)		154 (93%)		
Age (mean ± SD)		63.5 ± 11.2		68.3 ± 12.4		63.1 ± 11.0		*p* = 0.924
ASA	1	1	0.6%	0	0.0%	1	0.6%	*p* = 0.149
	2	71	42.8%	2	16.7%	69	44.8%	
	3	89	53.6%	9	75.0%	80	51.9%	
	4	5	3%	1	8.3%	4	2.6%	
BMI [kg/m^2^] (mean ± SD)		26.7 ± 5.2		28.0 ± 5.3		26.6 ± 5.2		*p* = 0.816
BMI [kg/m^2^]	<18.5	5	3%	0	0%	5	3.2%	*p* = 0.038
	18.5–24.9	63	38%	2	16.7%	61	39.6%	
	25–29.9	58	34.9%	9	75%	49	31.8%	
	≥30	40	24.1%	1	8.3%	39	25.3%	
pT	pT1a	12	7.5%	0	0%	12	7.9%	*p* = 0.380
	pT1b	28	17.4%	2	20%	26	17.2%	
	pT2	24	14.9%	4	40%	20	13.2%	
	pT3	67	41.6%	3	30%	64	42.4%	
	pT4a	3	1.9%	0	0%	3	2%	
	pT0	27	16.8%	1	10%	26	17.2%	
pN	pN1	27	16.3%	3	25%	24	15.6%	*p* = 0.040
	pN2	21	12.7%	4	33.3%	17	11%	
	pN3	26	15.7%	2	16.7%	24	15.6%	
	pN0	92	55.4%	3	25%	89	57.8%	
pM	pM1	7	4.2%	0	0%	7	4.5%	*p* = 1
	pM0	159	95.8%	12	100%	147	95.5%	
Positive LN (mean ± SD)		2.8 ± 5.2		3.6 ± 5.0		2.8 ± 5.2		*p* = 0.962
R	R1	10	6%	0	0%	10	6.5%	*p* = 1
	R0	156	94%	12	100%	144	93.5%	
R1 location	none	156	94%	12	100%	144	93.5%	*p* = 1
	circumferential	8	4.8%	0	0%	8	5.2%	
	distal	1	0.6%	0	0%	1	0.6%	
	proximal	1	0.6%	0	0%	1	0.6%	
Histology	Adenocarcinoma	133	80.1%	10	83.3%	123	79.9%	*p* = 1
	SCC	31	18.7%	2	16.7%	29	18.8%	
	Neuroendocrine	2	1.2%	0	0%	2	1.3%	
UICC	1a	12	7.2%	0	0%	12	7.8%	*p* = 0.324
	1b	26	15.7%	2	16.7%	24	15.6%	
	2	5	3%	1	8.3%	4	2.6%	
	2a	11	6.6%	1	8.3%	10	6.5%	
	2b	4	2.4%	0	0%	4	2.6%	
	3a	29	17.5%	3	25%	26	16.9%	
	3b	24	14.5%	2	16.7%	22	14.3%	
	4a	24	14.5%	3	25%	21	13.6%	
	9	7	4.2%	0	0%	7	4.5%	
	0	22	13.3%	0	0%	22	14.3%	
	none	2	1.2%	0	0%	2	1.3%	

ASA—American Society of Anesthesiologists classification; BMI—body mass index; LN—lymph nodes; SCC—squamous cell carcinoma; SD—standard deviation; WP—white plane.

**Table 2:** Peri- and postoperative complications in the patient collective stratified according to the Clavien–Dindo classification.

		Total		WP Not Visualized		WP Visualized		
		166		12		154		*p*
Clavien–Dindo (*n*, %)	Clavien–Dindo 1	16	9.6%	0	0.0%	16	10.4%	
	Clavien–Dindo 2	14	8.4%	1	8.3%	13	8.4%	
	Clavien–Dindo 3a	30	18.1%	2	16.7%	28	18.2%	
	Clavien–Dindo 3b	9	5.4%	1	8.3%	8	5.2%	*p* = 0.753
	Clavien–Dindo 4	7	4.2%	1	8.3%	6	3.9%	
	Clavien–Dindo 5	2	1.2%	0	0.0%	2	1.3%	
	No complications	88	53%	7	58.3%	81	52.6%	

**Table 3:** Peri- and postoperative complications in the patient collective.

		Total		WP Not Visualized		WP Visualized		
		166		12		154		*p*
Bleeding (*n*, %)	No	164	98.8%	12	100%	152	98.7%	*p* = 1
	Yes	2	1.2%	0	0.0%	2	1.3%	
Anastomotic leakage (*n*, %)	No	155	93.4%	12	100%	143	92.9%	*p* = 1
	Yes	11	6.6%	0	0.0%	11	7.1%	
Pneumonia (*n*, %)	No	136	81.9%	8	66.7%	128	83.1%	*p* = 0.233
	Yes	30	18.1%	4	33.3%	26	16.9%	
Pleural effusion (*n*, %)	No	157	94.6%	11	91.7%	146	94.8%	*p* = 0.500
	Yes	9	5.4%	1	8.3%	8	5.2%	
Pneumothorax (*n*, %)	No	163	98.2%	12	100%	151	98.1%	*p* = 1
	Yes	3	1.8%	0	0.0%	3	1.9%	
Pulmonary embolism (*n*, %)	No	164	98.8%	12	100%	152	98.7%	*p* = 1
	Yes	2	1.2%	0	0.0%	2	1.3%	
ARDS (*n*, %)	No	160	96.4%	12	100%	148	96.1%	*p* = 1
	Yes	6	3.6%	0	0.0%	6	3.9%	
RLN-Paralysis (*n*, %)	No	155	93.4%	12	100%	143	92.9%	*p* = 1
	Yes	11	6.6%	0	0.0%	11	7.1%	
Chylothorax (*n*, %)	No	156	94.0%	12	100%	144	93.5%	*p* = 1
	Yes	10	6.0%	0	0.0%	10	6.5%	

ARDS—acute respiratory distress syndrome; RLN—right laryngeal nerve; WP—white plane.

**Table 4:** Complication management (chylothorax) and in-hospital mortality.

		Total		WP Not Visualized		WP Visualized		*p*
		166		12		154		
Complication management	chest tube, low-fat diet	5	3.0%	0	0.0%	5	3.2%	*p* = 1
	chest tube, low-fat diet, pleurolysis, clipping the TD	1	0.6%	0	0.0%	1	0.6%	
	chest tube, low-fat diet, pleurolysis, clipping and embolization of the TD	1	0.6%	0	0.0%	1	0.6%	
	chest tube, low-fat diet, suturing the TD	1	0.6%	0	0.0%	1	0.6%	
	chest tube, low-fat diet, suturing, clipping and embolization of the TD	1	0.6%	0	0.0%	1	0.6%	
	chest tube, low-fat diet, embolization of the TD	1	0.6%	0	0.0%	1	0.6%	
	none	156	94%	12	100%	144	93.5%	
IHM 30	no	166	100%	12	100%	154	100%	-
IHM 90	no	164	98.8%	12	100%	152	98.7%	*p* = 1
	yes	2	1.2%	0	0.0%	2	1.3%	

IHM—in-hospital mortality; TD—thoracic duct; WP—white plane.

**Table 5:** Patient survival log-rank analysis.

Cox Regression: *n* = 166, R^2^ = 0.314, Model: *p* < 0.001	*p*-Wert	HR	95%-CI	95%-CI	Estimate	SE	z-Value	*n*
BMI [kg/m^2^]	0.694	1.012	0.953	1.076	0.012	0.031	0.393	166
White Plane	0.055	0.384	0.144	1.020	−0.958	0.499	−1.920	166
ASA	0.006	1.996	1.223	3.259	0.691	0.250	2.765	166
Pneumonia	0.127	1.709	0.859	3.402	0.536	0.351	1.527	166
pT ≥ 3	0.000	4.583	2.191	9.586	1.522	0.377	4.043	166
pN ≥ 2	0.007	2.329	1.258	4.314	0.846	0.314	2.689	166
Neoadjuvant Treatment	0.368	1.412	0.666	2.994	0.345	0.383	0.901	166
Adjuvant Treatment	0.001	0.372	0.205	0.678	−0.988	0.306	−3.234	166

Cox Regression: *n* = 166, R^2^ = 0.314, Model: *p* < 0.001. Accuracy = 0.70 [0.62–0.77], TP = 0.70 [0.62–0.77], TN = 0.70 [0.62–0.77], PPV = 0.70 [0.62–0.77], NPV = 0.67 [0.59–0.74]. ASA—American Society of Anesthesiologists classification; BMI—body mass index; HR—hazard ratio; CI—confidence interval; SE—standard error.

**Table 6:** Recurrence-free survival log-rank analysis.

Cox Regression: *n* = 166, R^2^ = 0.258, Model: *p* < 0.001	*p*-Wert	HR	95%-CI	95%-CI	Estimate	SE	z-Value	*n*
***BMI [kg/m^2^]***	0.168	1.040	0.984	1.100	0.039	0.029	1.379	166
***White Plane***	0.121	0.494	0.203	1.203	−0.705	0.454	1.552	166
***ASA***	0.661	1.121	0.672	1.871	0.115	0.261	0.438	166
***Pneumonia***	0.204	0.570	0.240	1.356	−0.561	0.442	1.271	166
***pT ≥ 3***	0.156	1.713	0.815	3.599	0.538	0.379	1.420	166
***pN ≥ 2***	0.000	4.409	2.149	9.046	1.484	0.367	4.046	166
***Neoadjuvant Treatment***	0.781	1.113	0.521	2.379	0.107	0.387	0.278	166
***Adjuvant Treatment***	0.003	0.422	0.239	0.745	−0.863	0.29	2.976	166

Cox Regression: *n* = 166, R^2^ = 0.076, Model: *p* = 0.0222. Accuracy = 0.70 [0.62–0.77], TP = 0.70 [0.62–0.77], TN = 0.70 [0.62–0.77], PPV = 0.70 [0.62–0.77], NPV = 0.66 [0.59–0.73]. ASA—American Society of Anesthesiologists classification; BMI—body mass index; HR—hazard ratio; CI—confidence interval; SE—standard error

## Data Availability

The data presented in this study are available from the corresponding author upon request due to privacy, legal, and ethical restrictions.
